# Genome-Wide Identification of the WRKY Gene Family in Mulberry and Screening of Consistently Drought-Responsive Candidates

**DOI:** 10.3390/genes17101212

**Published:** 2026-09-29

**Authors:** Jie Li, Weifen Liu, Lei Xia, Chunping Huang, Yuemei Chen, Xianyan Zou

**Affiliations:** College of Biology and Food Engineering, Qujing Normal University, Qujing 655011, China

**Keywords:** *M. alba* cv. Yunsang-2, WRKY transcription factor, genome-wide identification, drought stress, subcellular localization

## Abstract

Background: WRKY transcription factors are widely involved in plant responses to drought. Mulberry (*Morus alba*) is an economically important tree in Southwest China, yet its WRKY gene family has not been systematically characterized at the genome level. Methods: In this study, we identified the WRKY gene family in mulberry by integrating bioinformatic analysis with transcriptome data and experimental verification. Results: A total of 56 MnWRKY genes were identified and classified into three major groups (I–III), with group II further divided into five subgroups (IIa–IIe). Promoter analysis detected 577 cis-acting elements belonging to ten representative categories, among which ABA-responsive elements were the most abundant. Chromosomal localization showed that 73.2% of the genes were concentrated on the first three chromosomes, and collinearity analysis identified three tandem and 21 segmental duplication pairs. By integrating public RNA-seq data with our transcriptome data from the drought-tolerant cultivar Yunsang-2, we found eight *MnWRKY* genes (*MnWRKY4*, *MnWRKY8*, *MnWRKY9*, *MnWRKY11*, *MnWRKY13*, *MnWRKY18*, *MnWRKY20*, and *MnWRKY21*) that were consistently upregulated under drought in both datasets. qRT-PCR further confirmed that *MnWRKY9* and *MnWRKY18* were significantly induced by drought, with *MnWRKY9* showing the strongest response. Transient expression in tobacco leaves showed that MnWRKY9 localizes to the nucleus. Conclusions: MnWRKY9 is identified as a candidate regulator of drought response in mulberry. These results provide a useful resource for further functional studies of WRKY genes and for molecular breeding of drought-tolerant mulberry varieties.

## 1. Introduction

Drought stress is one of the most severe environmental factors limiting plant growth and agricultural productivity under global climate change [1,2]. Plants have evolved complex molecular regulatory networks to cope with drought stress, among which transcription factor-mediated transcriptional reprogramming plays a central role in drought responses [3]. The WRKY transcription factor family is one of the largest transcription factor families in plants. Its members contain one or two highly conserved WRKY domains and can specifically bind to W-box elements (C/TTGACC/T) in the promoters of downstream target genes, thereby activating or repressing their transcription [4,5,6]. Based on the number of WRKY domains and the type of zinc-finger motif, the WRKY family is classified into three major groups: Group I (two WRKY domains with an C_2_H_2_ zinc-finger), Group II (one WRKY domain with an C_2_H_2_ zinc-finger), and Group III (one WRKY domain with an C_2_HC zinc-finger), with Group II further divided into five subgroups, IIa–IIe [4,6].

Since the first WRKY gene, *SPF1*, was identified in sweet potato [7], the drought-responsive functions of numerous WRKY transcription factors have been characterized in diverse plant species. In Group I, *Arabidopsis AtWRKY1* enhances drought tolerance by regulating stomatal movement [8], wheat *TaWRKY2* overexpression significantly improves grain yield under drought stress in transgenic wheat [9], and sweet potato *IbWRKY2* overexpression increases drought and salt tolerance in transgenic *Arabidopsis* through enhanced antioxidant enzyme activity and osmolyte accumulation [10]. In Group II, maize *ZmWRKY79* positively regulates drought tolerance by promoting ABA biosynthesis [11]. In Group III, *Arabidopsis AtWRKY46*, *AtWRKY54*, and *AtWRKY70* mediate drought tolerance through the brassinosteroid signaling pathway [12]. These studies demonstrate that WRKY transcription factors from different subfamilies participate in drought responses through diverse mechanisms, and that their functions exhibit a certain degree of evolutionary conservation, providing a theoretical basis for screening drought-responsive candidate genes from specific subfamilies.

Mulberry (*M. alba*) is an economically important tree species widely cultivated in Yunnan, Guangxi, and other regions of China, primarily for sericulture, with its fruits also possessing edible and medicinal value. Mulberry exhibits considerable environmental adaptability, tolerating drought, low temperature, and high temperature to a certain extent; however, seasonal drought remains a major factor limiting leaf yield and quality. In recent years, progress has been made in understanding the molecular mechanisms underlying drought stress responses in mulberry. Li et al. (2022) identified *MaWRKYIII8* as being upregulated by drought and ABA through combined transcriptomic and proteomic analyses, and classified it into Group III by phylogenetic analysis [13]; however, this study only performed a preliminary analysis of a single gene and did not conduct genome-wide identification and systematic classification of the mulberry WRKY family. Subsequently, our group used the Yunnan local cultivar Yunsang-2, a cultivar of *M. alba*, to reveal the roles of ABA, GA, and BR hormone signaling pathways in mulberry drought response through physiological and transcriptomic analyses, and annotated 11 WRKY members among the differentially expressed genes by aligning them against the PlantTFDB database [14], further suggesting the involvement of this family in mulberry drought response. Nevertheless, none of the above studies have performed a genome-wide systematic characterization of the WRKY gene family in mulberry. The gene complement, phylogenetic classification, protein structural features, chromosomal distribution, expansion mechanisms, and expression patterns under drought stress of this family remain unclear.

Therefore, in this study, we performed a genome-wide identification of the WRKY transcription factor family in mulberry, and systematically analyzed their phylogenetic relationships, conserved motifs, gene structures, promoter cis-acting elements, chromosomal localization, and collinearity relationships. Meanwhile, by integrating public transcriptome data and Yunsang-2 transcriptome data, we employed a dual-transcriptome integrative screening strategy to identify conserved upregulated WRKY candidate genes under drought stress, followed by qRT-PCR validation. Furthermore, subcellular localization analysis was conducted on the priority candidate gene *MnWRKY9* to verify the fundamental prerequisite for its presumed regulatory function as a nuclear-localized transcription factor. This study aimed to characterize the mulberry WRKY family at the genome level and to identify candidate genes that may contribute to drought tolerance in mulberry.

## 2. Results

### 2.1. Identification and Physicochemical Properties of MnWRKY Proteins

A total of 56 non-redundant WRKY genes were identified in the mulberry genome using the *Morus notabilis* reference through BLASTP and HMM searches (Table S1). The identified genes were designated *MnWRKY1* to *MnWRKY56* according to their chromosomal positions. The amino acid lengths of MnWRKY proteins ranged from 137 aa (MnWRKY8) to 823 aa (MnWRKY28), with molecular weights ranging from 15.75 to 90.29 kDa. The theoretical isoelectric point (pI) varied from 4.93 (MnWRKY41) to 9.68 (MnWRKY23), and 71.43% (40/56) of the predicted proteins had a pI below 7, indicating a predominance of acidic proteins. The instability index ranged from 36.86 (MnWRKY48) to 68.64 (MnWRKY8). Only two proteins, MnWRKY14 (38.54) and MnWRKY48 (36.86), had an instability index below 40, suggesting that they may be stable, whereas the remaining 54 members are predicted to be unstable. The aliphatic index ranged from 39.53 to 80.19, and the grand average of hydropathicity (GRAVY) ranged from −1.171 to −0.265. All MnWRKY proteins had negative GRAVY values, indicating that they are hydrophilic. Subcellular localization prediction using WoLF PSORT showed that 94.6% (53/56) of the MnWRKY proteins were predicted to localize predominantly in the nucleus, with 15 members, including MnWRKY9 and MnWRKY18, receiving the maximal nuclear localization score (nucl: 14), strongly suggesting that they are nuclear proteins. These results show considerable physicochemical diversity among the MnWRKY transcription factors, which may be associated with their diverse functions in different biological processes.

### 2.2. Phylogenetic Analysis of the MnWRKY Family

**Figure 1:**
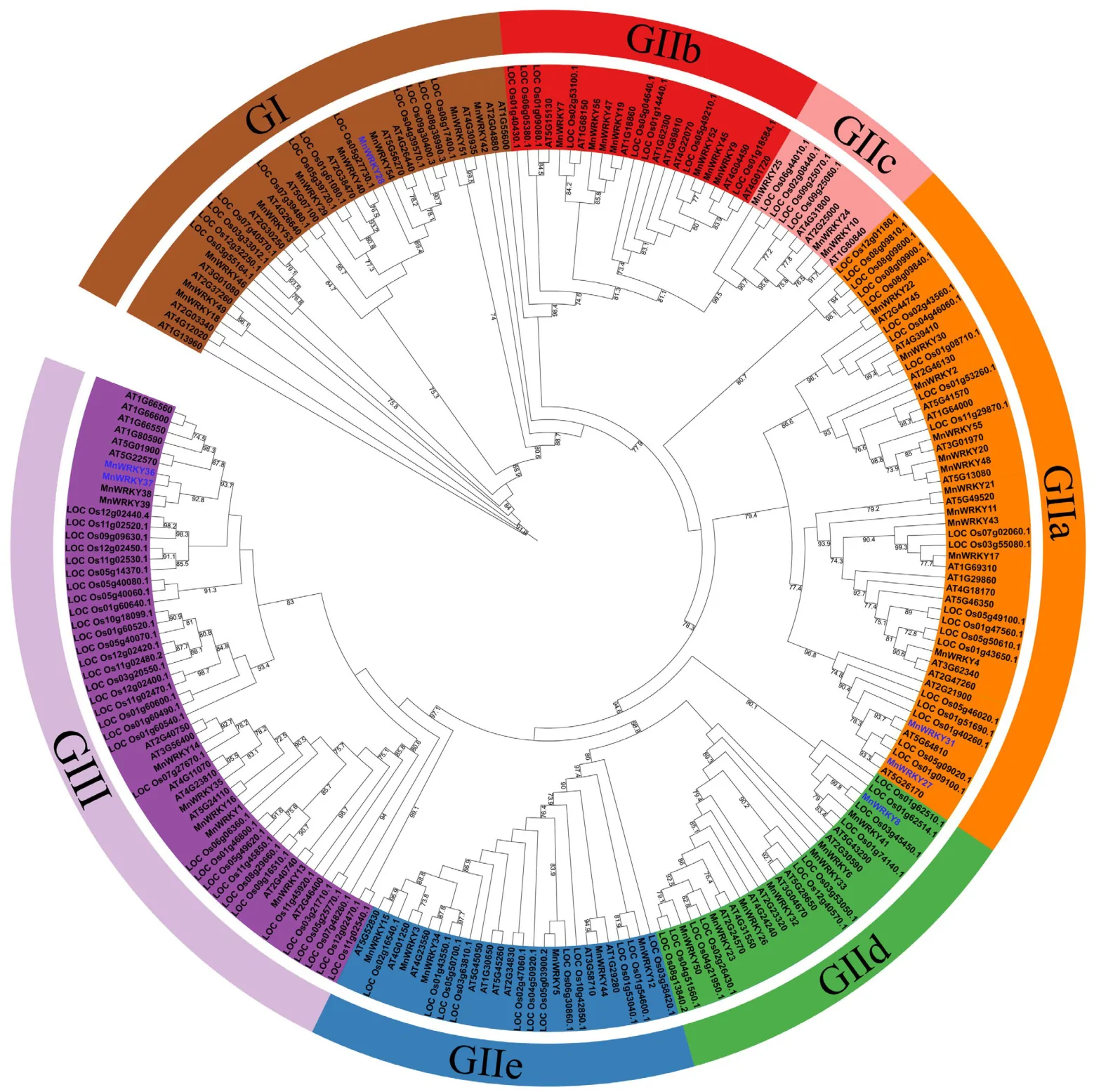
Maximum likelihood phylogenetic tree of WRKY proteins from *M. notabilis*, *A. thaliana*, and *O. sativa*. The tree was constructed using IQ-TREE v3.1.3 with the best-fit model Q.PLANT + I + R5 selected by ModelFinder. One WRKY domain per protein was used; for Group I proteins, the C-terminal domain was retained. Bootstrap values (1000 ultrafast replicates) ≥ 50% are shown at the nodes. MnWRKY proteins with atypical or partial WRKY domains are labelled in green.

To examine the evolutionary relationships of MnWRKY proteins, a maximum likelihood phylogenetic tree was constructed using WRKY domain sequences from mulberry, *Arabidopsis thaliana*, and *Oryza sativa* (Figure 1). The 56 MnWRKY proteins were classified into three major groups: Group I (10 members), Group II (37 members), and Group III (9 members). Group II was further divided into five subgroups: IIa (13 members), IIb (7 members), IIc (3 members), IId (8 members), and IIe (6 members). Subgroup IIa was the largest and IIc the smallest. According to the phylogenetic analysis, MnWRKY9 was assigned to subgroup IId, while MnWRKY18 belonged to Group I. Notably, both clades contain multiple previously characterized drought-responsive regulators, suggesting that they may be involved in drought responses, although functional validation is required. The WRKY domain alignment showed that all MnWRKY proteins contain the conserved WRKYGQK heptapeptide or its variants, together with the C_2_H_2_/C_2_HC zinc-finger motif (Figure S1). In addition to the canonical WRKYGQK, atypical variants such as WRKYGKK and WKKYGQK were identified in MnWRKY proteins, and these variants have also been reported in other plant species, including *Arabidopsis*. MnWRKY proteins with atypical or partial WRKY domains are labelled in green in Figure 1.

### 2.3. Conserved Motifs and Gene Structure Analysis

**Figure 2:**
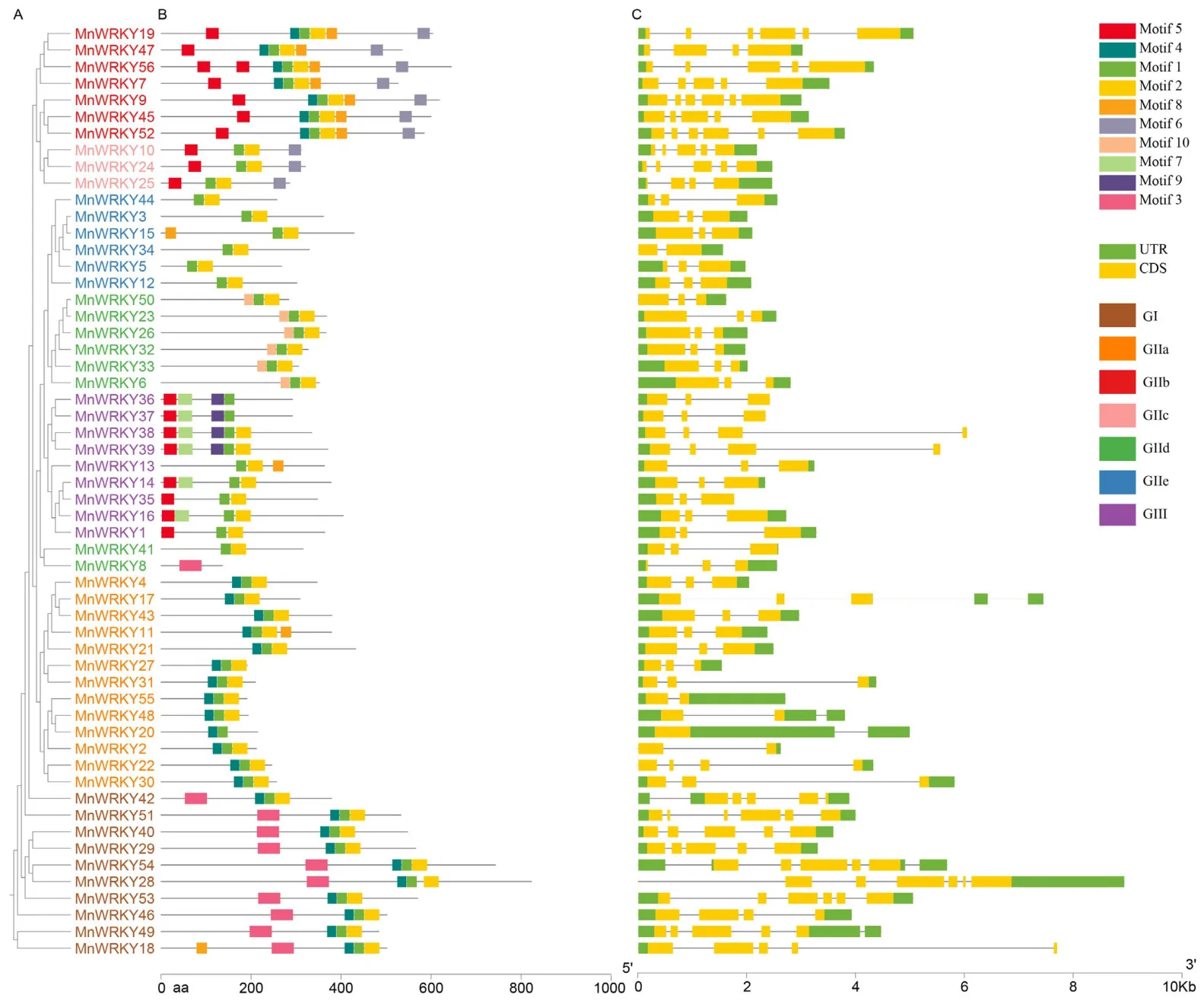
Conserved motifs and gene structure of MnWRKY proteins. (**A**) Maximum likelihood phylogenetic tree of the 56 MnWRKY proteins. (**B**) Distribution of 10 conserved motifs identified by MEME. Each colored box represents a distinct motif, with box sizes proportional to motif length. (**C**) Exon–intron organization of *MnWRKY* genes. Yellow boxes indicate coding sequences (CDSs), black lines represent introns, and green boxes indicate untranslated regions (UTRs).

To further characterize the structural features of MnWRKY proteins, conserved motif and gene structure analyses were performed (Figure 2). A total of 10 conserved motifs were identified. Motifs 1 and 2, which represent the core WRKY domain, were widely distributed across most family members (Figure S2). Motif 3 was exclusively present in all Group I members, consistent with the dual-WRKY-domain architecture characteristic of this group. Motifs 8 and 10 were enriched in Group III proteins. Within Group II, subgroup IIb specifically contained motifs 4, 6, and 7, which were absent or rare in other subgroups. These subgroup-specific motif patterns support the phylogenetic classification and suggest potentially conserved functions within each clade.

Gene structure analysis revealed that the number of exons in *MnWRKY* genes ranged from one to six. Group I genes generally possessed five to six exons, Group II genes contained two to five exons, and Group III genes predominantly had three to four exons. Notably, *MnWRKY36* and *MnWRKY37* exhibited nearly identical exon–intron structures, differing only in the length of their 5′-UTRs, consistent with their origin from a tandem duplication event.

### 2.4. Chromosomal Localization and Duplication Events

**Figure 3:**
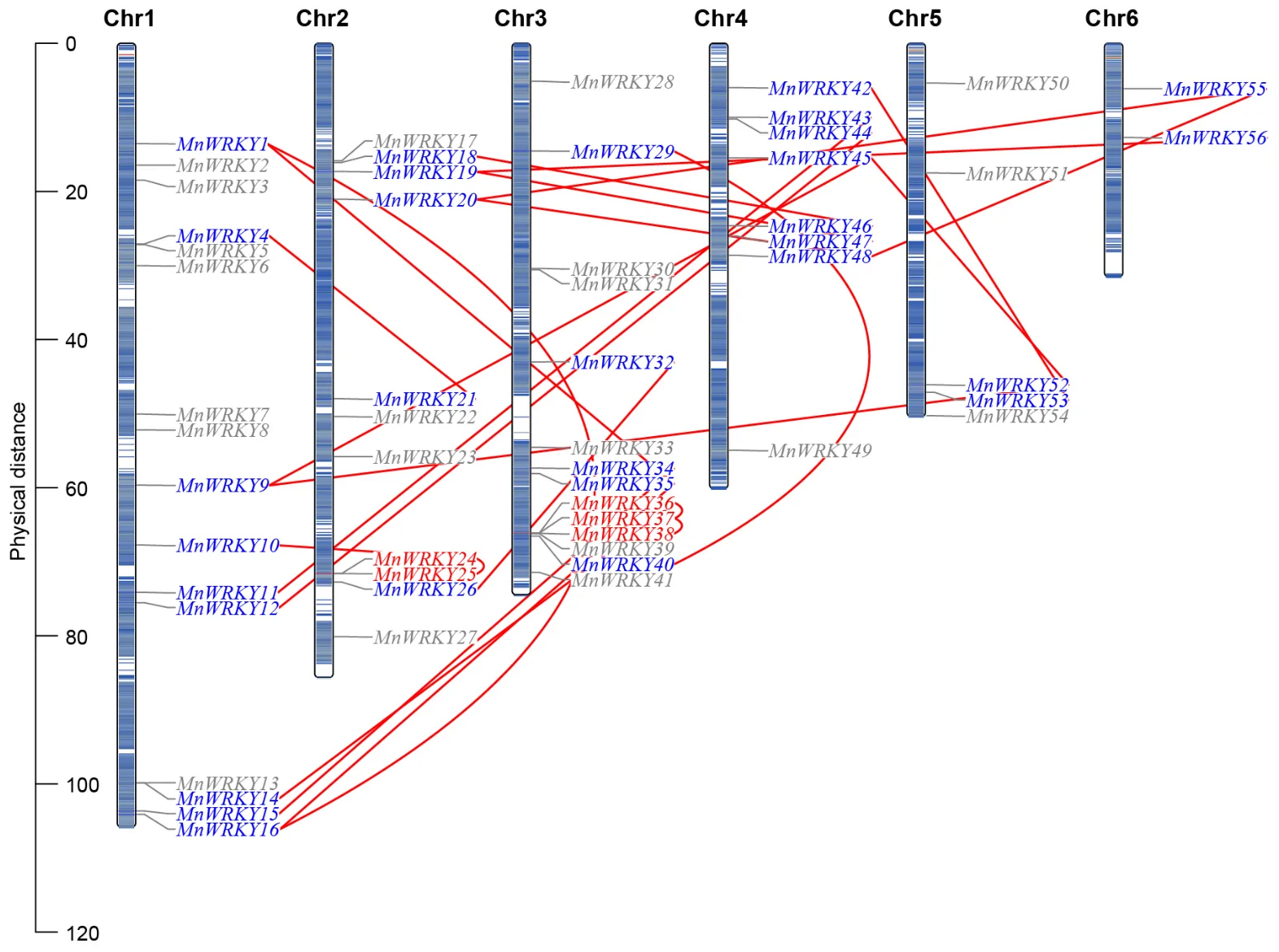
Chromosomal localization and tandem duplication analysis of MnWRKY genes. The six *M. notabilis* chromosomes (Chr1–Chr6) are displayed with the physical positions of MnWRKY genes. The grayscale bar on each chromosome indicates gene density. Red lines connect tandemly duplicated genes. Three tandem duplication pairs were identified, involving three gene pairs: *MnWRKY24*/*MnWRKY25* on Chr2, and *MnWRKY36*/*MnWRKY37* and *MnWRKY37*/*MnWRKY38* on Chr3.

The 56 *MnWRKY* genes were unevenly distributed across all six mulberry chromosomes (Figure 3). The majority were concentrated on chromosome 1 (16 genes, 28.6%), chromosome 2 (11 genes, 19.6%), and chromosome 3 (14 genes, 25%), collectively accounting for 73.2% (41/56) of all family members. Chromosomes 4, 5, and 6 harbored 8, 5, and 2 genes, respectively.

**Figure 4:**
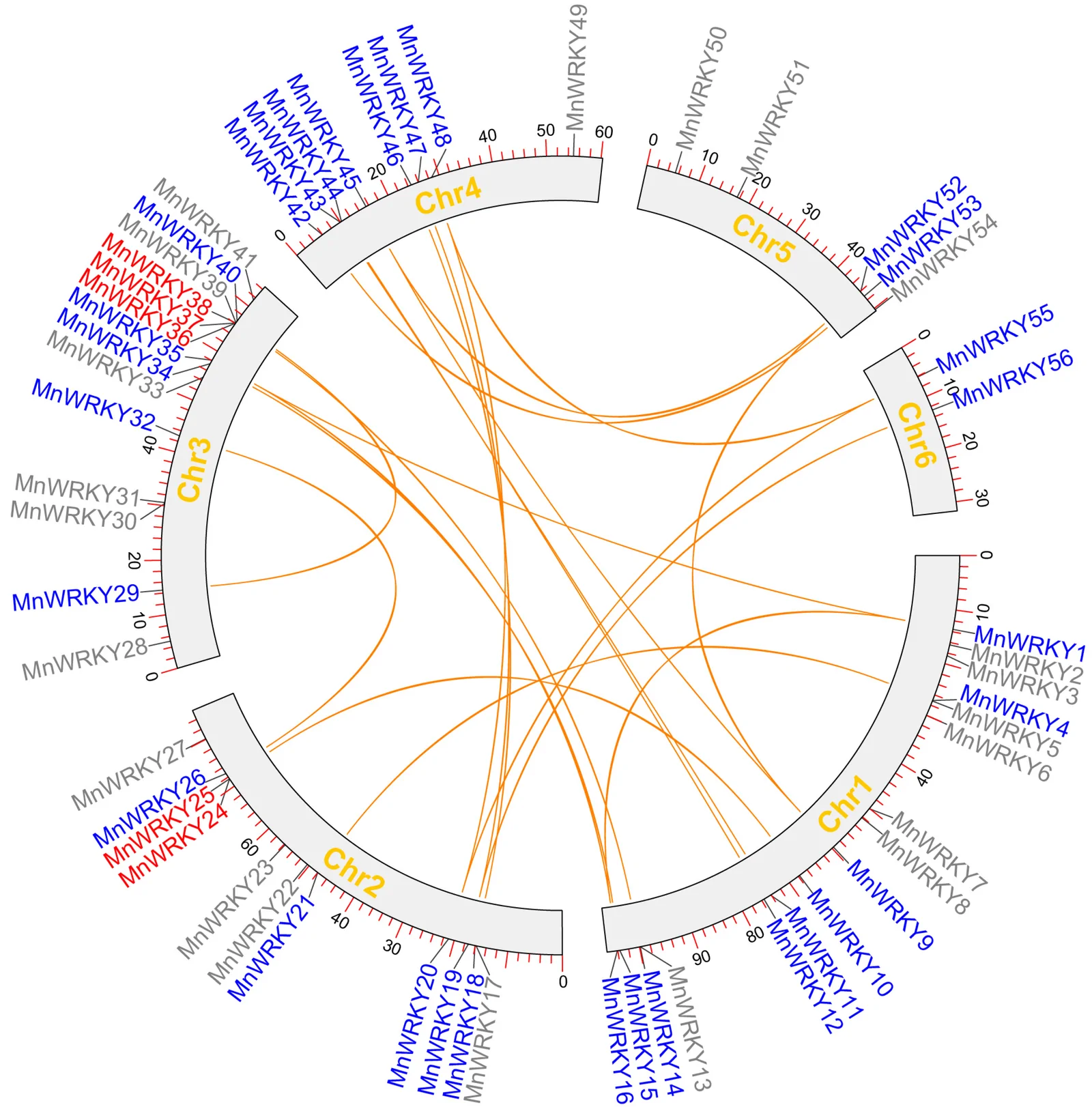
Intragenomic collinearity analysis of *MnWRKY* genes. The circular map displays the six *M. notabilis* chromosomes. Colored curved lines connect the 21 segmentally duplicated gene pairs listed in Table S2; representative pairs include *MnWRKY1*/*MnWRKY16*, *MnWRKY18*/*MnWRKY46*, *MnWRKY19*/*MnWRKY47*, and *MnWRKY20*/*MnWRKY48*.

Collinearity analysis identified 24 duplicated MnWRKY gene pairs, including three tandem duplication pairs (MnWRKY24/MnWRKY25, MnWRKY36/MnWRKY37, and MnWRKY37/MnWRKY38) and 21 segmental duplication pairs (Figure 4, Table S2). Ka/Ks analysis showed that all pairs with calculable values had Ka/Ks < 1, indicating purifying selection. Notably, the segmentally duplicated pair MnWRKY18/MnWRKY46 had a Ka/Ks ratio of 0.187, suggesting that their coding sequences have remained relatively conserved. The tandem pair MnWRKY36/MnWRKY37 showed Ks = 0 and an extremely low Ka, consistent with a very recent duplication event. These duplication events have contributed to the expansion of the MnWRKY gene family. To examine the evolutionary conservation of WRKY genes between mulberry and *Arabidopsis*, we performed an interspecies collinearity analysis. A total of 58 orthologous WRKY gene pairs were identified between the two species, and these pairs are shown in Figure S3.

### 2.5. Cis-Acting Element Analysis of MnWRKY Gene Promoters

**Figure 5:**
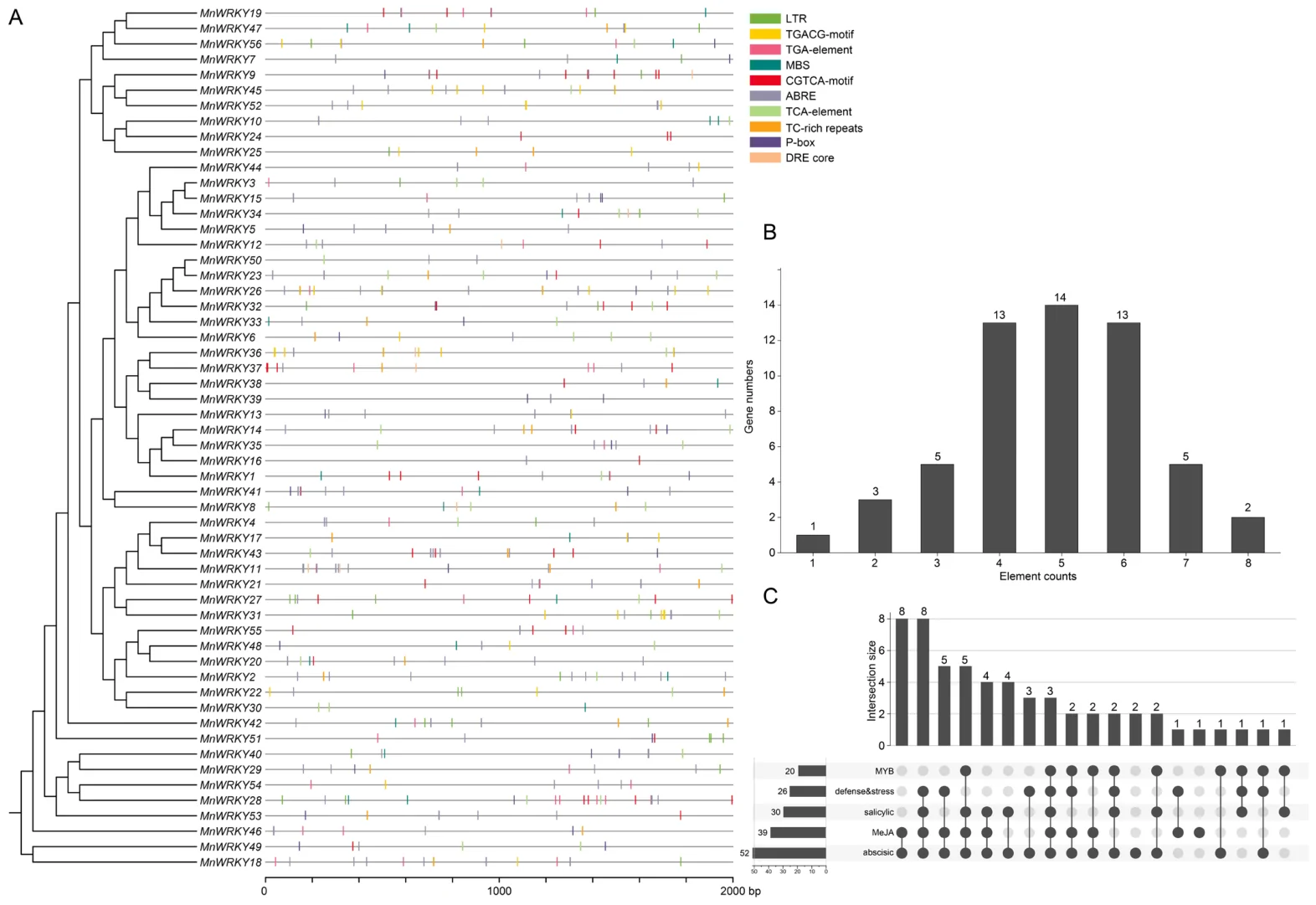
Cis-acting regulatory element analysis of *MnWRKY* gene promoters. (**A**) Distribution of ten representative types of cis-acting elements in the 2000-bp upstream promoter regions of 56 *MnWRKY* genes. (**B**) Bar chart showing the number of genes containing each cis-element type. (**C**) UpSet plot illustrating the co-occurrence patterns of five major stress-responsive cis-elements. ABRE, abscisic acid-responsive element; MBS, MYB binding site involved in drought inducibility; CGTCA-motif and TGACG-motif, MeJA-responsive elements; TCA-element, salicylic acid-responsive element; TC-rich repeats, defense and stress-responsive element; LTR, low-temperature-responsive element; P-box, gibberellin-responsive element; TGA-element, auxin-responsive element; DRE core, dehydration-responsive element.

To investigate the potential regulatory mechanisms of *MnWRKY* genes, cis-acting elements within the 2000-bp upstream promoter regions of all 56 genes were analyzed using PlantCARE (Figure 5). Multiple types of cis-acting regulatory elements were identified. Ten representative stress-, hormone-, and development-related element categories were selected for visualization. ABA-responsive elements (ABREs) were the most abundant, with 190 elements distributed across 52 gene promoters. MeJA-responsive elements (CGTCA-motif and TGACG-motif) were present in 39 genes (185 elements), SA-responsive elements (TCA-element) in 30 genes (42 elements), and defense/stress-responsive TC-rich repeats in 26 genes (30 elements). Low-temperature-responsive elements (LTR) were detected in 22 genes (33 elements). In addition, 23 drought-inducible MYB binding sites (MBSs), 35 auxin-responsive elements (TGA-element), 31 gibberellin-responsive elements (P-box), and 8 dehydration-responsive elements (DRE core) were identified. In total, 577 stress- and hormone-responsive cis-elements were found, suggesting that MnWRKY transcription factors may be subject to regulation by multiple hormone signaling pathways in stress responses. To further explore the relationship between promoter composition and drought-responsive expression, we compared the cis-element profiles of the eight consistently drought-responsive genes with those of the remaining *MnWRKY* genes. The eight genes showed higher average numbers of ABREs (5.62 vs. 2.92) and AREs (3.00 vs. 1.80) than the other genes (Figure S4). Notably, *MnWRKY9* was enriched in MeJA-responsive elements, whereas *MnWRKY18* was enriched in ABREs, suggesting that they may be regulated through different hormone signaling pathways.

### 2.6. Expression Analysis of MnWRKY Genes Under Drought Stress

#### 2.6.1. Transcriptomic Profiling

**Figure 6:**
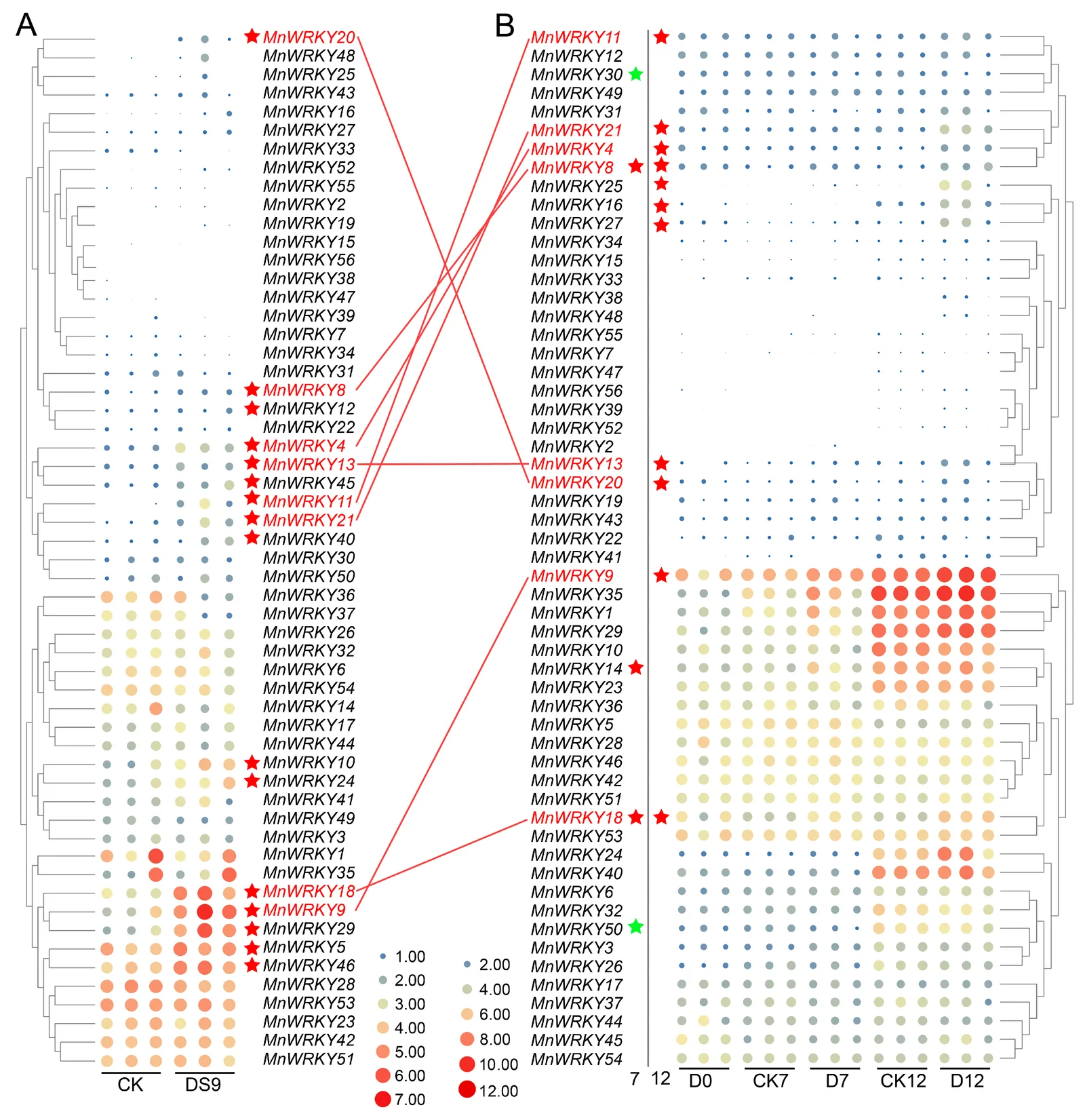
Expression heatmaps of *MnWRKY* genes under drought stress from two independent transcriptome datasets. (**A**) Public RNA-seq data (PRJNA692033) from mulberry leaves after 9 days of drought treatment. (**B**) RNA-seq data from mulberry cv. Yunsang-2 leaves under drought stress at 7 and 12 days post-stress (DPS). FPKM values were Z-score normalized and log_2_-transformed. Red and green asterisks denote significantly up- and down-regulated genes, respectively (|log_2_ fold change| > 1, FDR < 0.05). Red lines connect the eight *MnWRKY* genes (*MnWRKY4*, *MnWRKY8*, *MnWRKY9*, *MnWRKY11*, *MnWRKY13*, *MnWRKY18*, *MnWRKY20*, and *MnWRKY21*) that were commonly and significantly upregulated in both datasets, representing a core set of consistently drought-responsive candidates.

To investigate the expression patterns of *MnWRKY* genes under drought stress, we analyzed two independent transcriptome datasets. Re-analysis of the public RNA-seq data (PRJNA692033; drought 9 days vs. control) identified 16 significantly upregulated *MnWRKY* genes (|log_2_FC| > 1, FDR < 0.05) (Figure 6A). In the Yunsang-2 transcriptome dataset, 3 and 2 genes were significantly up- and down-regulated at 7 DPS, respectively, while 11 genes were significantly upregulated at 12 DPS (Figure 6B). Among them, *MnWRKY8* and *MnWRKY18* were significantly upregulated at both 7 and 12 DPS in Yunsang-2.

To identify drought-responsive *MnWRKY* genes that were consistently upregulated across two independent datasets, we overlapped the significantly upregulated genes from the two independent datasets. Eight genes—*MnWRKY4*, *MnWRKY8*, *MnWRKY9*, *MnWRKY11*, *MnWRKY13*, *MnWRKY18*, *MnWRKY20*, and *MnWRKY21*—were commonly upregulated, representing a core set of consistently drought-responsive candidate WRKY genes in mulberry (Figure 6).

#### 2.6.2. qRT-PCR Validation

**Figure 7:**
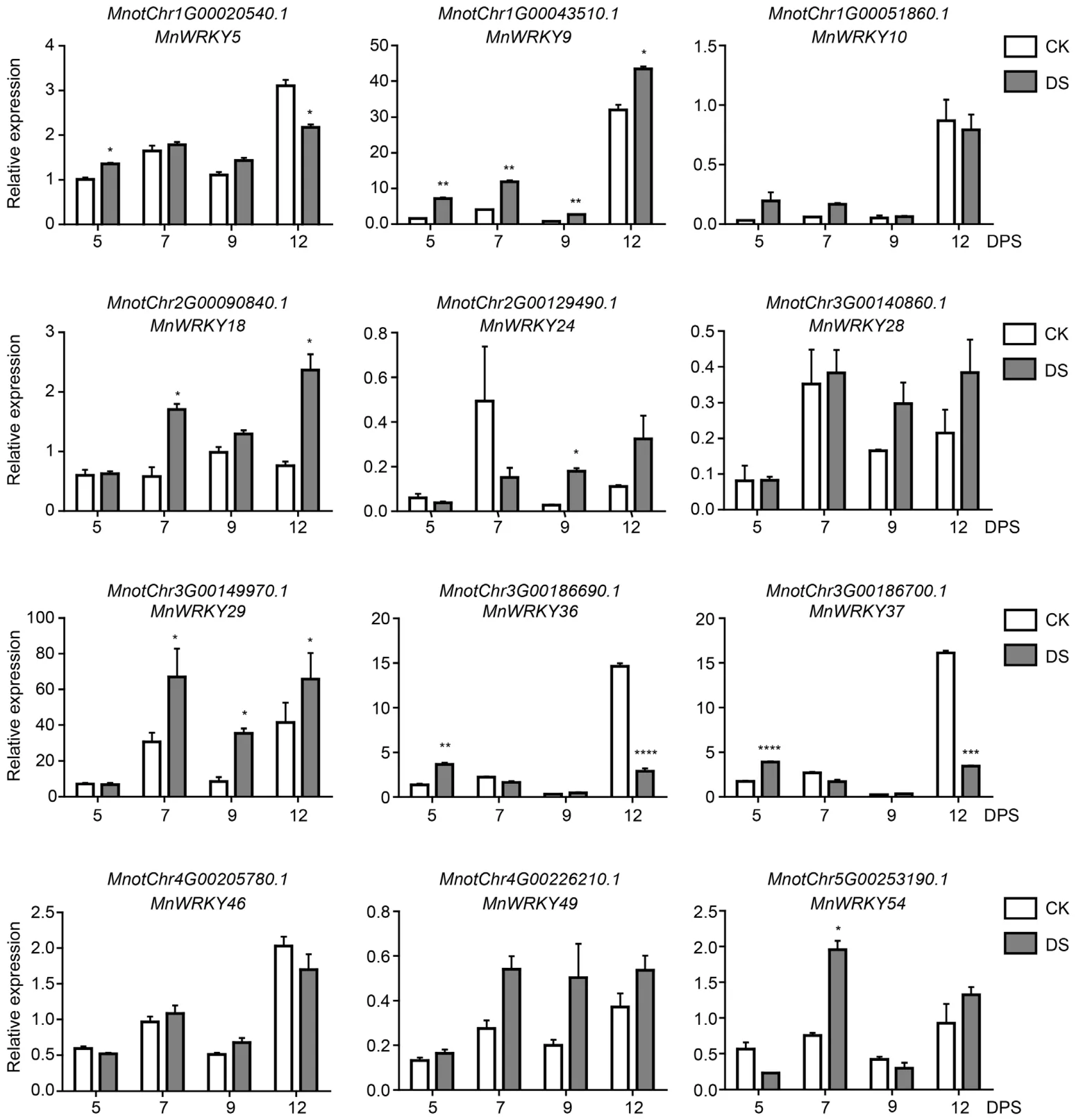
Quantitative real-time PCR (qRT-PCR) validation of selected *MnWRKY* genes under progressive drought stress. Total RNA was extracted from *M. notabilis* cv. Yunsang-2 leaves at 5, 7, 9, and 12 DPS. *MnACTIN7* was used as the internal reference gene. Relative expression was calculated using the 2^−ΔΔCt^ method. Data represent mean ± SD from three biological replicates. Asterisks indicate statistically significant differences compared with the control (*, *p* < 0.05; **, *p* < 0.01; ***, *p* < 0.001; ****, *p* < 0.0001; two-way ANOVA followed by Sidak’s multiple comparison test).

To validate the transcriptomic results, we initially designed primers for approximately 20 *MnWRKY* genes that showed significant differential expression in the public RNA-seq dataset (PRJNA692033; Li et al., 2022 [13]). Among them, 12 genes with satisfactory amplification efficiency and melt curves were selected for qRT-PCR analysis across a progressive drought time course (0, 5, 7, 9, and 12 DPS) in Yunsang-2 (Figure 7). After our Yunsang-2 transcriptome data became available (Li et al., 2026 [14]), we performed a cross-dataset comparison and identified eight genes that were consistently upregulated in both datasets. Among these eight genes, *MnWRKY9* and *MnWRKY18* were already included in the 12 qRT-PCR-validated genes, and both showed sustained and significant upregulation under drought stress, consistent with the RNA-seq data. The remaining six genes were not included in the qRT-PCR validation because the primer design and validation experiments were completed before the cross-dataset comparison was performed.

Among the tested genes, *MnWRKY24* showed a slight upregulation at 12 DPS by qRT-PCR, but this change did not reach statistical significance, which was concordant with its non-significant expression change in the Yunsang-2 RNA-seq data. The tandemly duplicated genes *MnWRKY36* and *MnWRKY37* exhibited a downward trend in RNA-seq that did not reach statistical significance; however, in the qRT-PCR assay, they were significantly downregulated at the later drought stage (12 DPS). Notably, the segmentally duplicated gene pair *MnWRKY18*/*MnWRKY46* displayed divergent expression patterns under drought stress: *MnWRKY18* was consistently induced in both transcriptomes, whereas *MnWRKY46* was significantly upregulated only in the public dataset and showed no significant change in Yunsang-2. These results suggest that MnWRKY transcription factors are involved in drought stress responses, and that the expression patterns of some *MnWRKY* genes may vary among different mulberry cultivars.

#### 2.6.3. Subcellular Localization Assay of MnWRKY9

**Figure 8:**
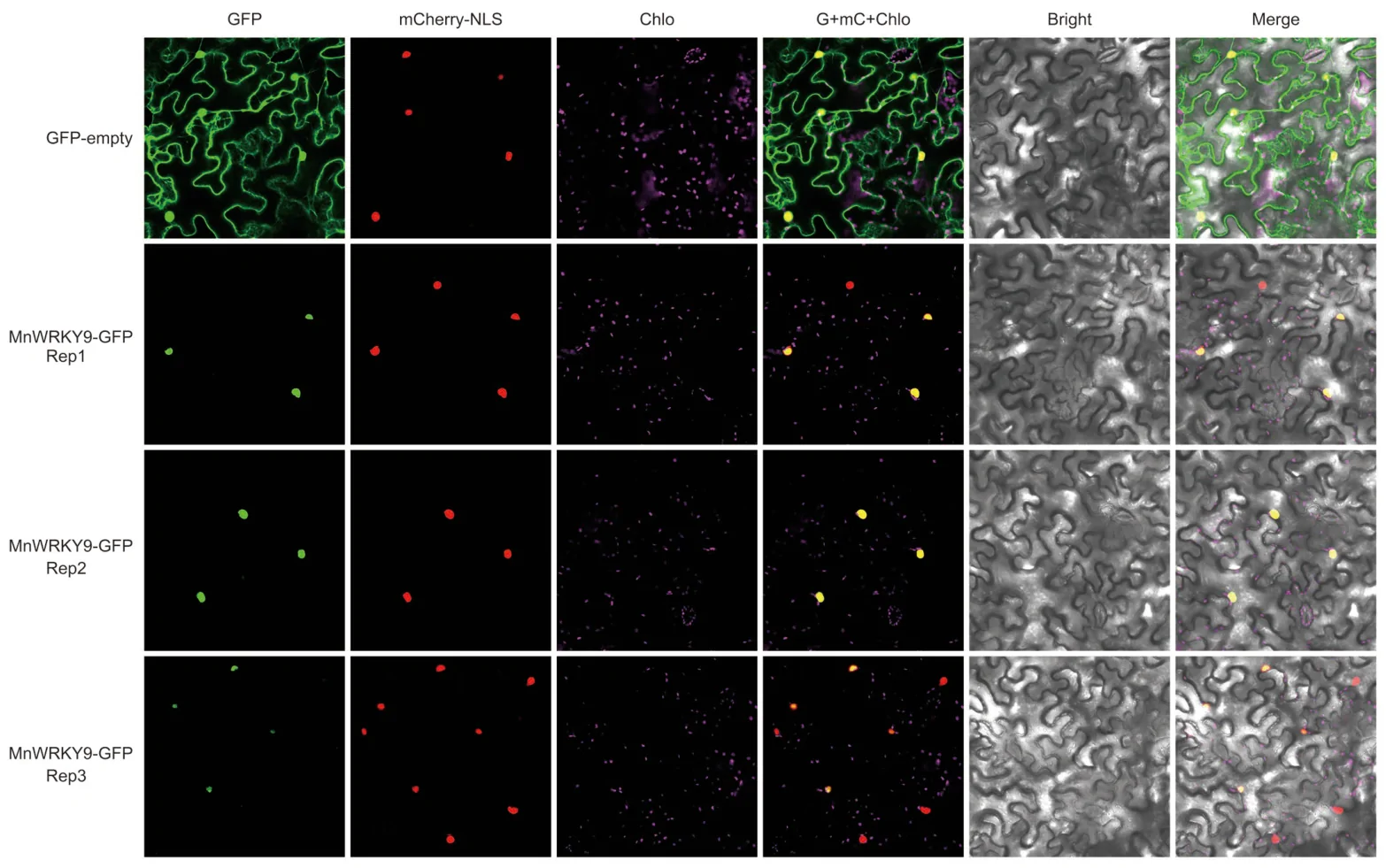
Subcellular localization of MnWRKY9. The *35S:MnWRKY9-GFP* fusion protein and the free GFP control were transiently expressed in *N. benthamiana* leaves together with an mCherry-NLS nuclear marker. Fluorescence signals were observed using confocal laser scanning microscopy. Green fluorescence represents the GFP signal, red fluorescence represents the mCherry-NLS nuclear marker, and magenta represents chloroplast autofluorescence. The merged images show the co-localization of MnWRKY9-GFP with the nucleus. Three independent experiments were performed with similar results. Representative images from one experiment are shown. In total, at least 10 cells with detectable GFP signal were examined across the three replicates. Scale bars = 20 μm.

Among the two qRT-PCR-confirmed candidate genes, MnWRKY9 was prioritized for subcellular localization analysis based on its robust and sustained induction pattern and its clear assignment to the functionally characterized IId subgroup. WoLF PSORT prediction assigned MnWRKY9 a maximal nuclear localization score (nucl: 14). To experimentally verify this prediction, the *35S:MnWRKY9-GFP* fusion construct and the free GFP control were transiently expressed in *Nicotiana benthamiana* leaves. Confocal microscopy revealed that the green fluorescence of the MnWRKY9-GFP fusion protein was exclusively confined to the nucleus, where it colocalized precisely with the mCherry-NLS nuclear marker signal and did not overlap with chloroplast autofluorescence, whereas the fluorescence of free GFP was distributed throughout the cytoplasm and nucleus (Figure 8). This result demonstrates that MnWRKY9 is a nuclear-localized protein, confirming the computational prediction for MnWRKY9. Three independent experiments were performed with similar results. In total, at least 10 cells with detectable GFP signal were examined across the three replicates, and MnWRKY9-GFP was predominantly localized to the nucleus in all examined cells. However, nuclear localization of the remaining family members still requires experimental verification.

## 3. Discussion

Drought stress is a major environmental factor limiting mulberry growth under global climate change. Yunnan Province, a key region for sericulture in China, experiences frequent seasonal drought, making the identification of drought-responsive genes in Yunnan-adapted cultivars particularly important. Previously, using the local cultivar Yunsang-2, we demonstrated that drought stress significantly increased CAT and POD activities as well as proline accumulation in leaves, and transcriptomic analysis identified 11 WRKY members among the differentially expressed genes [14], suggesting the involvement of this family in mulberry drought response. Although Li et al. (2022) performed a preliminary characterization of the mulberry WRKY family and reported that *MaWRKYIII8* was induced by drought and ABA [13], their study did not include a genome-wide analysis covering systematic classification, protein structural features, chromosomal distribution, and synteny relationships, nor did it evaluate the conservation of drought responses across different cultivars. In this study, we therefore performed genome-wide identification, dual-transcriptome integrative screening, qRT-PCR validation, and subcellular localization assays to systematically characterize the sequence features and evolutionary patterns of the MnWRKY family and to identify key candidates that may play central regulatory roles in drought stress responses.

Through HMM profiling combined with BLASTP homology searches, we identified a total of 56 MnWRKY transcription factors in the mulberry genome. It should be noted that Yunsang-2 is a cultivar of *M. alba*, whereas the reference genome used here is *M. notabilis* [15]. Although the two species are closely related, using a heterologous reference genome may lead to incomplete gene models or missed genes. Compared with the 11 WRKY members previously obtained by aligning differentially expressed genes against the PlantTFDB database [14], the de novo identification strategy employed in this study is more comprehensive. Re-analysis of the Yunsang-2 transcriptome data using this expanded gene set detected 13 differentially expressed *MnWRKY* genes under drought stress (11 upregulated and 2 downregulated). This methodological improvement ensures that downstream expression analysis and candidate gene screening are based on a more complete family gene set. Phylogenetic analysis classified these 56 members into the canonical three major groups (I, II, and III), with group II further subdivided into five subgroups (IIa–IIe), a pattern highly consistent with that reported in *Arabidopsis*, rice, and many other plant species [4,6]. This cross-species conservation indicates that the core WRKY subfamilies were largely established before the divergence of monocots and dicots and retained in the mulberry genome. Within the family, MnWRKY9 was assigned to subgroup IId, while MnWRKY18 belongs to group I. Accumulating evidence has shown that members of both clades are widely involved in drought stress responses. In group I, overexpression of wheat *TaWRKY2* and sweet potato *IbWRKY2* significantly enhanced drought tolerance in transgenic plants [9,10]. In subgroup IId, silencing of cotton *Gh_A11G1801* increased drought sensitivity [16], whereas heterologous overexpression of *MfWRKY7* from the resurrection plant *Myrothamnus flabellifolia* and *PgWRKY44* from pearl millet improved drought and salt tolerance in *Arabidopsis* [17,18]. These findings from diverse species suggest that group I and subgroup IId may share conserved drought-responsive functions, although direct functional evidence in mulberry is still lacking. Conserved motif and gene structure analyses further confirmed the intra-subgroup similarity, supporting the reliability of the phylogenetic grouping. Additionally, both tandem and segmental duplications contributed to the expansion of the *MnWRKY* family. Ka/Ks analysis of the duplicated pairs showed that they have been subject to purifying selection, suggesting that these duplicated genes have retained conserved functions during evolution. Notably, the segmentally duplicated gene pair *MnWRKY18*/*MnWRKY46* displayed divergent expression patterns under drought: *MnWRKY18* was consistently induced by drought in both independent transcriptomes and qRT-PCR, whereas *MnWRKY46* was significantly upregulated only in the public transcriptome dataset but not in Yunsang-2. The Ka/Ks ratio of this pair was 0.187, indicating purifying selection on the coding sequences, suggesting that the divergent expression patterns are more likely attributable to regulatory divergence rather than coding-sequence divergence. Based on these findings, we propose that *MnWRKY18*/*MnWRKY46* may be undergoing subfunctionalization at the regulatory level, although this hypothesis requires further experimental validation. This expression divergence may be attributable to differences in the promoter cis-element composition, genomic position effects, or epigenetic modifications between the duplicate copies. The underlying mechanisms, however, require further investigation through promoter sequence alignment and functional experiments.

To reduce potential false positives arising from cultivar- or condition-specific variation in single-transcriptome analyses, we integrated two independent drought transcriptome datasets: the public dataset (PRJNA692033, 9 days post-drought) [13] and the Yunsang-2 dataset (7 and 12 days post-drought) [14]. By overlapping the significantly upregulated genes from both datasets, we identified these eight genes as consistently upregulated across different genotypes and drought durations. This dual-transcriptome cross-validation strategy enhanced the reliability of the candidate genes. Subsequent qRT-PCR analysis further narrowed the focus to *MnWRKY9* and *MnWRKY18*, both of which displayed consistent drought-induced upregulation across different platforms and time points, with *MnWRKY*9 showing particularly robust and sustained induction. In contrast, the tandemly duplicated genes *MnWRKY36* and *MnWRKY37* showed a non-significant downward trend in RNA-seq but reached statistical significance in qRT-PCR at the later drought stage, highlighting the influence of detection platform sensitivity and regulatory hierarchy within gene families, and underscoring the importance of integrating multiple methods in candidate gene screening. Notably, Li et al. (2022) previously reported that *MaWRKYIII8* (corresponding to *MnWRKY35* in our study based on sequence alignment) was upregulated under drought and ABA treatments [13]. However, *MnWRKY35* was not significantly differentially expressed in either our re-analysis of their dataset or in the Yunsang-2 transcriptome data (Figure 6). This discrepancy is most likely attributable to differences in genome annotation version, alignment pipeline, and plant cultivar, rather than to an error in gene identification. BLASTP analysis confirmed that MaWRKYIII8 (XP_010104968.1) and MnWRKY35 (MnotChr3G00179840.1) share 100% sequence identity (Figure S5 and Table S3). This observation further supports the necessity of adopting a multi-cultivar cross-validation strategy to identify consistently drought-responsive candidates across two independent datasets.

As a transcription factor, nuclear localization is a prerequisite for its regulatory function. WoLF PSORT predicted a maximal nuclear localization score (nucl: 14) for MnWRKY9, and this prediction was fully corroborated by our transient expression assay in tobacco leaves: the green fluorescence of the MnWRKY9-GFP fusion protein precisely colocalized with the mCherry-NLS nuclear marker signal and was distinct from chloroplast autofluorescence, whereas free GFP was distributed throughout the cell. This result provides experimental evidence that MnWRKY9 is a nuclear-localized protein, confirming the computational prediction. However, nuclear localization of the remaining family members still requires experimental verification. Among the two qRT-PCR-confirmed candidate genes, we prioritized *MnWRKY9* for subcellular localization analysis based on its higher induction level and its clear assignment to the functionally characterized IId subgroup; experimental verification of the nuclear localization of MnWRKY18, which also received a maximal prediction score, awaits future studies.

Taken together, the phylogenetic placement within drought-responsive clades, the consistent upregulation across multiple datasets, and the experimentally confirmed nuclear localization collectively establish *MnWRKY9* as a core candidate regulator in mulberry drought response. We hypothesize that drought signals activate the transcription of *MnWRKY9*, and the translated MnWRKY9 protein translocates into the nucleus, where it may bind to W-box elements in the promoters of downstream target genes. However, the functions of IId subgroup members are diverse: cotton *GhWRKY21* (IId) negatively regulates drought tolerance by modulating ABA signaling [19], and several *Arabidopsis* IId WRKYs form transcriptional repressor complexes with OBERON proteins to balance growth and stress tolerance [20]. Therefore, whether MnWRKY9 acts as an activator or a repressor in mulberry remains to be determined through transgenic approaches, and this model requires functional validation. Furthermore, MnWRKY18, which was also validated and belongs to group I which harbors several well-characterized drought regulators, merits parallel functional investigation. Promoter analysis revealed that MnWRKY genes are enriched in ABA-, SA-, MeJA-, and drought-inducible cis-elements, suggesting that they may be subject to regulation by multiple hormone signaling pathways. However, the functional relevance of these elements remains to be experimentally verified. Future studies will focus on heterologous overexpression, identification of downstream target genes, and CRISPR/Cas9-based gene editing in mulberry to dissect the biological functions of *MnWRKY9* and *MnWRKY18*, thereby providing theoretical foundations and gene resources for molecular breeding of drought-tolerant mulberry varieties.

## 4. Conclusions

In this study, 56 WRKY genes were systematically identified in the *M. notabilis* genome. Phylogenetic, structural, and chromosomal analyses revealed their classification, conserved features, and duplication-driven expansion. By integrating dual-transcriptome cross-validation with time-course qRT-PCR, *MnWRKY9* and *MnWRKY18* were identified as core consistently drought-responsive candidates. The nuclear localization of MnWRKY9 was experimentally confirmed, supporting its potential regulatory role. These findings provide a basis for further functional dissection of WRKY-mediated drought response and offer valuable gene targets for the molecular breeding of drought-resistant mulberry varieties.

## 5. Materials and Methods

### 5.1. Plant Materials and Drought Treatment

The mulberry cultivar Yunsang-2 used in this study is a cultivar of *M. alba*, as described in our previous study [14]. Briefly, seedlings of Yunsang-2 were grown under normal management in a greenhouse. Drought stress was initiated by withholding water. Leaf samples were collected at 0, 5, 7, 9, and 12 days post-stress (DPS), immediately frozen in liquid nitrogen, and stored at −80 °C until use. Control plants were watered normally once per week.

### 5.2. Identification of the WRKY Gene Family in M. notabilis

The *M. notabilis* genome was used as the reference for read mapping and gene prediction, and the identified genes were designated with the *Mn* prefix accordingly [15]. The genome sequence and annotation files of *M. notabilis* were downloaded from the National Genomics Data Center (https://ngdc.cncb.ac.cn/gwh/Assembly/37805/show (accessed on 12 June 2024)). Genome sequences and annotation files of *A. thaliana* and *O. sativa* were obtained from Phytozome (https://phytozome-next.jgi.doe.gov/ (accessed on 12 June 2024)). *Arabidopsis* WRKY protein sequences were downloaded from the TAIR database (https://www.arabidopsis.org (accessed on 12 June 2024)). BLASTP (version 2.13.0+) searches were performed using Arabidopsis WRKY proteins as queries against mulberry and rice protein sequences (e-value < 1 × 10^−5^). In parallel, the WRKY domain HMM profile (PF03106) from Pfam was used for HMMsearch (version 3.3.2) against the same protein sequences with the --cut_ga parameter. The intersection of BLASTP and HMMsearch results was retained, and redundant transcripts were removed. The resulting candidate WRKY proteins were designated MnWRKY1–MnWRKY56 according to their chromosomal positions.

### 5.3. Physicochemical Property Analysis and Subcellular Localization Prediction

Physicochemical properties of the 56 MnWRKY proteins, including amino acid length, molecular weight (MW), theoretical isoelectric point (pI), instability index, aliphatic index, and grand average of hydropathicity (GRAVY), were analyzed using the ProtParam tool on the ExPASy server (https://www.expasy.org/ (accessed on 12 June 2024)). Subcellular localization was predicted using the WoLF PSORT online tool (https://wolfpsort.hgc.jp/ (accessed on 12 June 2024)) [21].

### 5.4. Phylogenetic Analysis

WRKY domain sequences were extracted using hmmsearch (HMMER v3.4) against the Pfam WRKY domain model (PF03106) with the gathering threshold. For proteins containing two WRKY domains (Group I), only the C-terminal domain was retained, because the C-terminal domain is homologous to the single WRKY domain present in Groups II and III and is commonly used for family-wide phylogenetic comparison. The extracted domain sequences were aligned using MAFFT v7.526 with the --auto option. The maximum likelihood (ML) tree was constructed using IQ-TREE v3.1.3. The best-fit amino acid substitution model was automatically selected by ModelFinder according to the Bayesian Information Criterion (BIC), and the ML tree was inferred with 1000 ultrafast bootstrap replicates. The resulting tree was visualized using iTOL.

### 5.5. Conserved Motif and Gene Structure Analysis

Conserved motifs of MnWRKY proteins were identified using the MEME online tool (https://meme-suite.org/meme/tools/meme (accessed on 12 June 2024)) with the number of motifs set to 10 and all other parameters as default. Gene structure information was extracted from the mulberry genome annotation files. The phylogenetic tree, conserved motif distribution, and gene structure were visualized using TBtools-II.

### 5.6. Promoter Cis-Acting Element Analysis

The upstream 2000-bp sequences of all *M. notabilis* genes were extracted from the genome and annotation files using TBtools-II. Promoter sequences of the 56 *MnWRKY* genes were subsequently retrieved by gene ID and submitted to the PlantCARE online tool (https://bioinformatics.psb.ugent.be/webtools/plantcare/html/ (accessed on 12 June 2024)) for cis-acting regulatory element identification. The results were visualized using TBtools-II. An upset plot of selected cis-element categories was generated using the Chiplot online tool (https://www.chiplot.online/index.html (accessed on 12 June 2024)).

### 5.7. Chromosomal Localization and Collinearity Analysis

Intraspecific BLASTP alignment of the M. notabilis protein sequences was performed using TBtools-II [22]. The resulting BLAST output was converted to MCScanX-compatible format, and the mulberry genome annotation GFF3 file was converted to the required GFF format. Both files were submitted to MCScanX [23] to generate tandem and collinearity output files. Tandem duplication information was extracted and visualized for chromosomal localization and tandem repeat analysis. Interchromosomal collinearity relationships were visualized using the Circle Gene View program in TBtools-II.

### 5.8. Public Transcriptome Data Analysis

Public RNA-seq data of mulberry leaves subjected to 9 days of drought stress (BioProject accession: PRJNA692033) were downloaded from the NCBI SRA database (https://www.ncbi.nlm.nih.gov/ (accessed on 12 June 2024)). Quality control was performed using fastp v1.3.7 [24] with default parameters. The *M. notabilis* genome index was built using HISAT2 v2.2.3 [25]. Sequence alignment, transcript assembly, and expression quantification were carried out using the HISAT2–StringTie–Ballgown pipeline [26], yielding FPKM expression values. A heatmap of *MnWRKY* gene expression was generated using TBtools-II. A count matrix was generated using the prepDE.py script, and differentially expressed genes (DEGs) under drought stress were identified using DESeq2 [27] with the criteria |log_2_ fold change| > 1 and FDR < 0.05.

### 5.9. Yunsang-2 Transcriptome Data Analysis

Transcriptome sequencing and analysis of Yunsang-2 under drought stress were performed in our previous study [14], which generated a complete gene expression matrix. In the present study, *MnWRKY* gene expression data were extracted from this matrix for downstream analysis. A detailed comparison of the two RNA-seq datasets, including biological replicates, sequencing depth, and mapping rates, is provided in Table S4.

### 5.10. RNA Extraction and cDNA Synthesis

Total RNA was extracted from frozen leaf samples collected at each time point using the Tiangen Total RNA Extraction Kit (DP419; Tiangen Biotech, Beijing, China) following the manufacturer’s instructions. RNA concentration was measured using a spectrophotometer, and all samples were adjusted to a uniform concentration. First-strand cDNA was synthesized using the TransScript cDNA Synthesis Kit (AE311; TransGen Biotech, Beijing, China) according to the manufacturer’s protocol.

### 5.11. qRT-PCR Primer Design and Analysis

qRT-PCR primers were obtained from the qPrimeDB database (https://biodb.org/), and their specificity was verified using NCBI Primer-BLAST. The verified primers were synthesized by Sangon Biotech (Shanghai, China). qRT-PCR was performed on a LightCycler 96 instrument (Roche, Basel, Switzerland) using PerfectStart Green qPCR SuperMix (TransGen Biotech, Beijing, China). Each 20 μL reaction was run with a two-step cycling program: 94 °C for 5 s and 60 °C for 30 s. Three biological replicates and three technical replicates were performed for each gene. Relative expression levels were calculated using the 2^−ΔΔCt^ method with *MnACTIN7* as the internal reference gene. Statistical significance was determined using two-way ANOVA followed by Sidak’s multiple comparison test, and bar charts were generated using GraphPad Prism 9.3. Primer sequences are listed in Table S5. The stability of *MnACTIN7* as an internal reference gene was validated using transcriptome data and compared with previously reported reference genes (Table S6). The coefficient of variation of candidate reference genes is shown in Figure S6.

### 5.12. Subcellular Localization of MnWRKY9

The full-length coding sequence (CDS) of *MnWRKY9* was amplified and cloned into the *pCAMBIA1300-GFP* vector to generate the *35S:MnWRKY9-GFP* fusion construct. The recombinant plasmid and the free GFP control (*pCAMBIA1300-GFP*) were separately transformed into Agrobacterium tumefaciens strain GV3101. Agrobacterium suspensions were infiltrated into the abaxial side of leaves of 4-week-old *N. benthamiana* plants [28]. The recombinant plasmid was co-infiltrated with an mCherry-NLS nuclear marker construct. After 48 h of incubation under normal growth conditions, leaf sections of appropriate size from the infiltrated area were excised. GFP fluorescence, mCherry fluorescence, and chloroplast autofluorescence were observed and photographed using a laser scanning confocal microscope (FV4000, Olympus, Tokyo, Japan). Three independent experiments were performed. In total, at least 10 cells with detectable GFP signal were examined across the three replicates. The GFP and mCherry channels were set at an excitation wavelength of 488 and 587 nm, respectively.

## Data Availability

The *M. notabilis* genome data are available from the National Genomics Data Center (NGDC, https://ngdc.cncb.ac.cn). The public RNA-seq data re-analyzed in this study are available from the NCBI SRA database under accession number PRJNA692033. The RNA-seq data of mulberry cv. Yunsang-2 under drought stress were generated by our group and have been deposited in the Genome Sequence Archive (GSA) under accession number CRA028403 (BioProject: PRJCA043810; https://ngdc.cncb.ac.cn/gsa/browse/CRA028403 (accessed on 12 June 2024)). All data generated or analyzed during this study are included in this published article and its Supplementary Materials.

## Supplementary Materials

The following supporting information can be downloaded at https://www.mdpi.com/article/10.3390/genes17101212/s1, Additional File S1: Table S1: Analysis of basic information of WRKY gene family members in *M. notabilis*; Table S2. Ka, Ks, and Ka/Ks ratios of tandem and segmental duplicated *MnWRKY* gene pairs; Table S3. BLASTP alignment of MaWRKYIII8 against the *M. notabilis* reference proteome; Table S4. Detailed information of the RNA-seq datasets used in this study; Table S5. Primers sequences for qRT-PCR in present study; Table S6. Stability of candidate reference genes under drought stress in mulberry. Additional File S2: Figure S1. Multiple sequence alignment of the WRKY domain in MnWRKY proteins; Figure S2. Sequence logos of the 10 conserved motifs identified in MnWRKY proteins; Figure S3. Interspecies collinearity analysis of WRKY genes between *A. thaliana* and *M. notabilis*; Figure S4. Cis-element analysis of the eight consistently drought-responsive MnWRKY genes; Figure S5. Pairwise sequence alignment of MaWRKYIII8 (XP_010104986.1) and MnWRKY35 (MnotChr3G00179840.1) from *M. notabilis*; Figure S6. Coefficient of variation (CV) of candidate reference genes under drought stress in mulberry.

## Abbreviations

The following abbreviations are used in this manuscript:

ABA	Abscisic acid
ABRE	ABA-responsive element
DPS	Days post-stress
DEG	Differentially expressed gene
FDR	False discovery rate
GRAVY	Grand average of hydropathicity
HMM	Hidden Markov Model
JA	Jasmonic acid
LTR	Low-temperature-responsive element
MeJA	Methyl jasmonate
MEME	Multiple Em for Motif Elicitation
NJ	Neighbor-joining
PlantCARE	Plant Cis-Acting Regulatory Element
qRT-PCR	Quantitative real-time polymerase chain reaction
SA	Salicylic acid
UTR	Untranslated region
Chlo	Chloroplast autofluorescence
GFP	Green fluorescent protein
mCherry-NLS	mCherry-tagged nuclear localization signal
NLS	Nuclear localization signal
ANOVA	analysis of variance
BIC	Bayesian information criterion
CDD	Conserved Domain Database
Ka/Ks	nonsynonymous (Ka)/synonymous (Ks) substitution rate ratio
ML	maximum likelihood
POD	peroxidase
SD	standard deviation
NGDC	National Genomics Data Center
pI	isoelectric point
RNA-seq	RNA sequencing

## References

[1] Liu S.J., Zhang H., Jin X.T., Niu M.X., Feng C.H., Liu X., Liu C., Wang H.L., Yin W., Xia X. (2025). PeFUS3 Drives Lateral Root Growth Via Auxin and ABA Signalling Under Drought Stress in Populus. Plant Cell Environ..

[2] Zhu J.-K. (2016). Abiotic stress signaling and responses in plants. Cell.

[3] Chen L., Song Y., Li S., Zhang L., Zou C., Yu D. (2012). The role of WRKY transcription factors in plant abiotic stresses. Biochim. Biophys. Acta (BBA) —Gene Regul. Mech..

[4] Eulgem T., Rushton P.J., Robatzek S., Somssich I.E. (2000). The WRKY superfamily of plant transcription factors. Trends Plant Sci..

[5] Ulker B., Somssich I.E. (2004). WRKY transcription factors: From DNA binding towards biological function. Curr. Opin. Plant Biol..

[6] Rushton P.J., Somssich I.E., Ringler P., Shen Q.J. (2010). WRKY transcription factors. Trends Plant Sci..

[7] Ishiguro S., Nakamura K. (1994). Characterization of a cDNA encoding a novel DNA-binding protein, SPF1, that recognizes SP8 sequences in the 5′ upstream regions of genes coding for sporamin and beta-amylase from sweet potato. Mol. General. Genet. MGG.

[8] Qiao Z., Li C.-L., Zhang W. (2016). WRKY1 regulates stomatal movement in drought-stressed Arabidopsis thaliana. Plant Mol. Biol..

[9] Gao H., Wang Y., Xu P., Zhang L., Li T., Zhao M., Li L. (2018). Overexpression of a WRKY transcription factor TaWRKY2 enhances drought stress tolerance in transgenic wheat. Front. Plant Sci..

[10] Zhu H., Zhou Y., Zhai H., He S., Zhao N., Liu Q. (2020). A Novel Sweetpotato WRKY Transcription Factor, IbWRKY2, Positively Regulates Drought and Salt Tolerance in Transgenic Arabidopsis. Biomolecules.

[11] Gulzar F., Fu J., Zhu C., Liu Y., Zhang M., Zhao Q., Li Z. (2021). Maize WRKY transcription factor ZmWRKY79 positively regulates drought tolerance through elevating ABA biosynthesis. Int. J. Mol. Sci..

[12] Chen J., Nolan T.M., Ye H., Zhang M., Tong H., Xin P., Chu J., Chu C., Li Z., Yin Y. (2017). Arabidopsis WRKY46, WRKY54, and WRKY70 transcription factors are involved in brassinosteroid-regulated plant growth and drought response. Plant Cell.

[13] Li R., Su X., Zhou R., Zhang Y., Wang T. (2022). Molecular mechanism of mulberry response to drought stress revealed by complementary transcriptomic and iTRAQ analyses. BMC Plant Biol..

[14] Li J., Deng Y., Ma Q., Liu W., Zou X. (2026). Comprehensive RNA-Seq analysis revealed molecular pathways and genes associated with drought tolerance in *Morus alba* cv. Yunsang-2. BMC Plant Biol..

[15] Ma B., Wang H., Liu J., Chen L., Xia X., Wei W., Yang Z., Yuan J., Luo Y., He N. (2023). The gap-free genome of mulberry elucidates the architecture and evolution of polycentric chromosomes. Hortic. Res..

[16] Gu L., Wang H., Wei H., Sun H., Li L., Chen P., Elasad M., Su Z., Zhang C., Ma L. (2018). Identification, Expression, and Functional Analysis of the Group IId WRKY Subfamily in Upland Cotton (*Gossypium hirsutum* L.). Front Plant Sci..

[17] Huang Z., Liu L., Jian L., Xu W., Wang J., Li Y., Jiang C.Z. (2022). Heterologous Expression of MfWRKY7 of Resurrection Plant Myrothamnus flabellifolia Enhances Salt and Drought Tolerance in Arabidopsis. Int. J. Mol. Sci..

[18] Saha B., Saha S., Kt M., Nayak J., Chanwala J., Dey N., Giri M.K. (2026). PgWRKY44-mediated modulation of SA/JA pathways enhances blast pathogen resistance in pearl millet and rice. Plant Signal Behav..

[19] Wang J., Wang L., Yan Y., Zhang S., Li H., Gao Z., Wang C., Guo X. (2021). GhWRKY21 regulates ABA-mediated drought tolerance by fine-tuning the expression of GhHAB in cotton. Plant Cell Rep..

[20] Du P., Wang Q., Yuan D.Y., Chen S.S., Su Y.N., Li L., Chen S., He X.J. (2023). WRKY transcription factors and OBERON histone-binding proteins form complexes to balance plant growth and stress tolerance. EMBO J..

[21] Horton P., Park K.J., Obayashi T., Fujita N., Harada H., Adams-Collier C.J., Nakai K. (2007). WoLF PSORT: Protein localization predictor. Nucleic Acids Res..

[22] Chen C., Wu Y., Li J., Wang X., Zeng Z., Xu J., Liu Y., Feng J., Chen H., He Y. (2023). TBtools-II: A “one for all, all for one” bioinformatics platform for biological big-data mining. Mol. Plant.

[23] Wang Y., Tang H., Debarry J.D., Tan X., Li J., Wang X., Lee T.H., Jin H., Marler B., Guo H. (2012). MCScanX: A toolkit for detection and evolutionary analysis of gene synteny and collinearity. Nucleic Acids Res..

[24] Chen S., Zhou Y., Chen Y., Gu J. (2018). fastp: An ultra-fast all-in-one FASTQ preprocessor. Bioinformatics.

[25] Kim D., Paggi J.M., Park C., Bennett C., Salzberg S.L. (2019). Graph-based genome alignment and genotyping with HISAT2 and HISAT-genotype. Nat. Biotechnol..

[26] Pertea M., Kim D., Pertea G.M., Leek J.T., Salzberg S.L. (2016). Transcript-level expression analysis of RNA-seq experiments with HISAT, StringTie and Ballgown. Nat. Protoc..

[27] Love M.I., Huber W., Anders S. (2014). Moderated estimation of fold change and dispersion for RNA-seq data with DESeq2. Genome Biol..

[28] Sparkes I.A., Runions J., Kearns A., Hawes C. (2006). Rapid, transient expression of fluorescent fusion proteins in tobacco plants and generation of stably transformed plants. Nat. Protoc..

