# Supplementary material for: Genome-Wide Identification of the WRKY Gene Family in Mulberry and Screening of Consistently Drought-Responsive Candidates
Source: Genes (Basel). 2026 Sep 29;17(10):1212. doi: 10.3390/genes17101212 (PMC13704373; doi:10.3390/genes17101212)
Supplement: Supplementary file 1 [file genes-17-01212-s001.zip › Additional file S2.pdf]

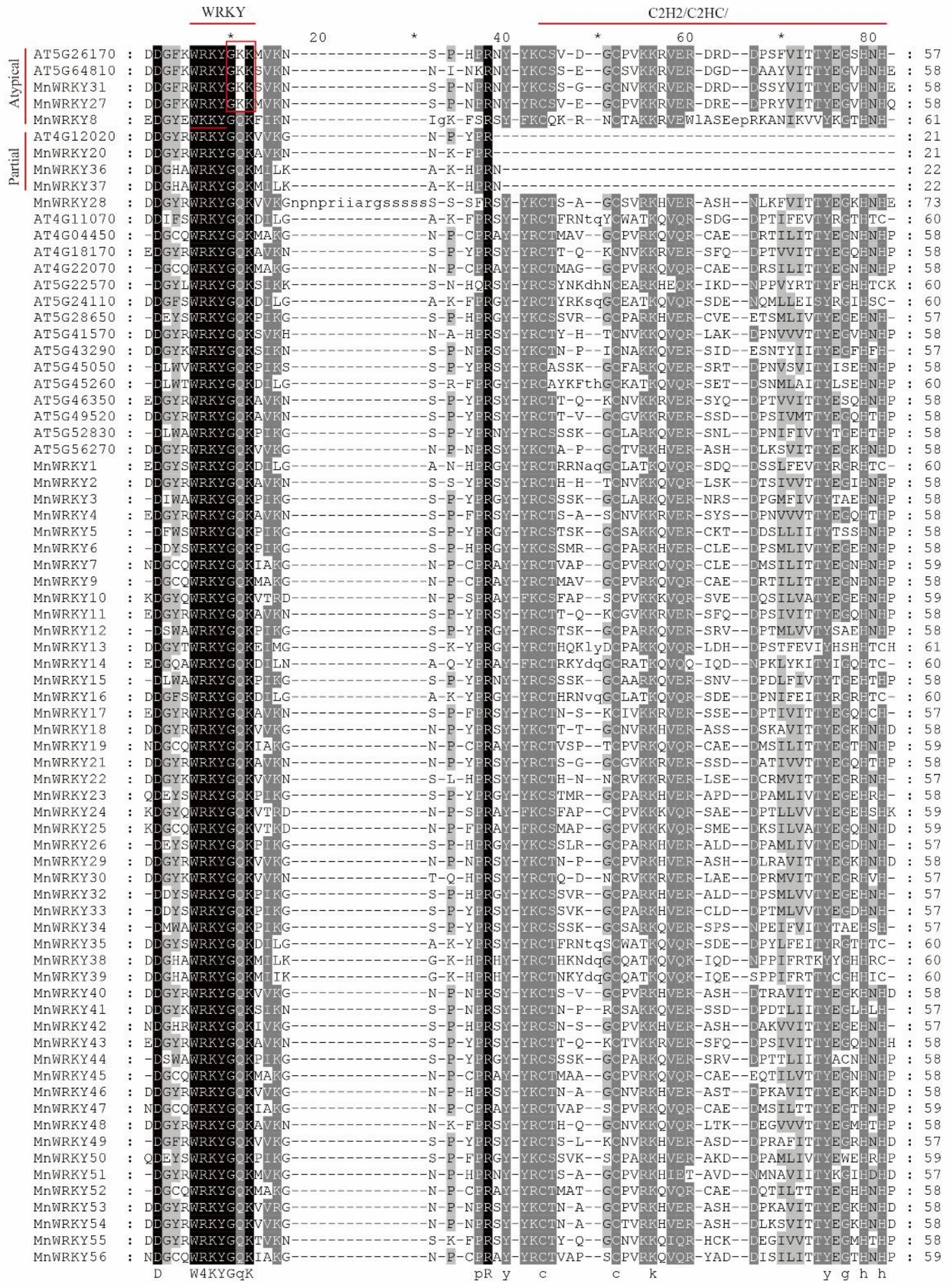

Supplementary Figure S1. Multiple sequence alignment of the WRKY domain in MnWRKY proteins.

Note: The alignment includes the WRKY domains of all 56 MnWRKY proteins and selected WRKY proteins from *A. thaliana*. Rice sequences were not included to focus on the structural features of the mulberry WRKY family. Red boxes and line indicate the atypical WRKY heptapeptide variants WRKYGKK and WKKYGQK, respectively.

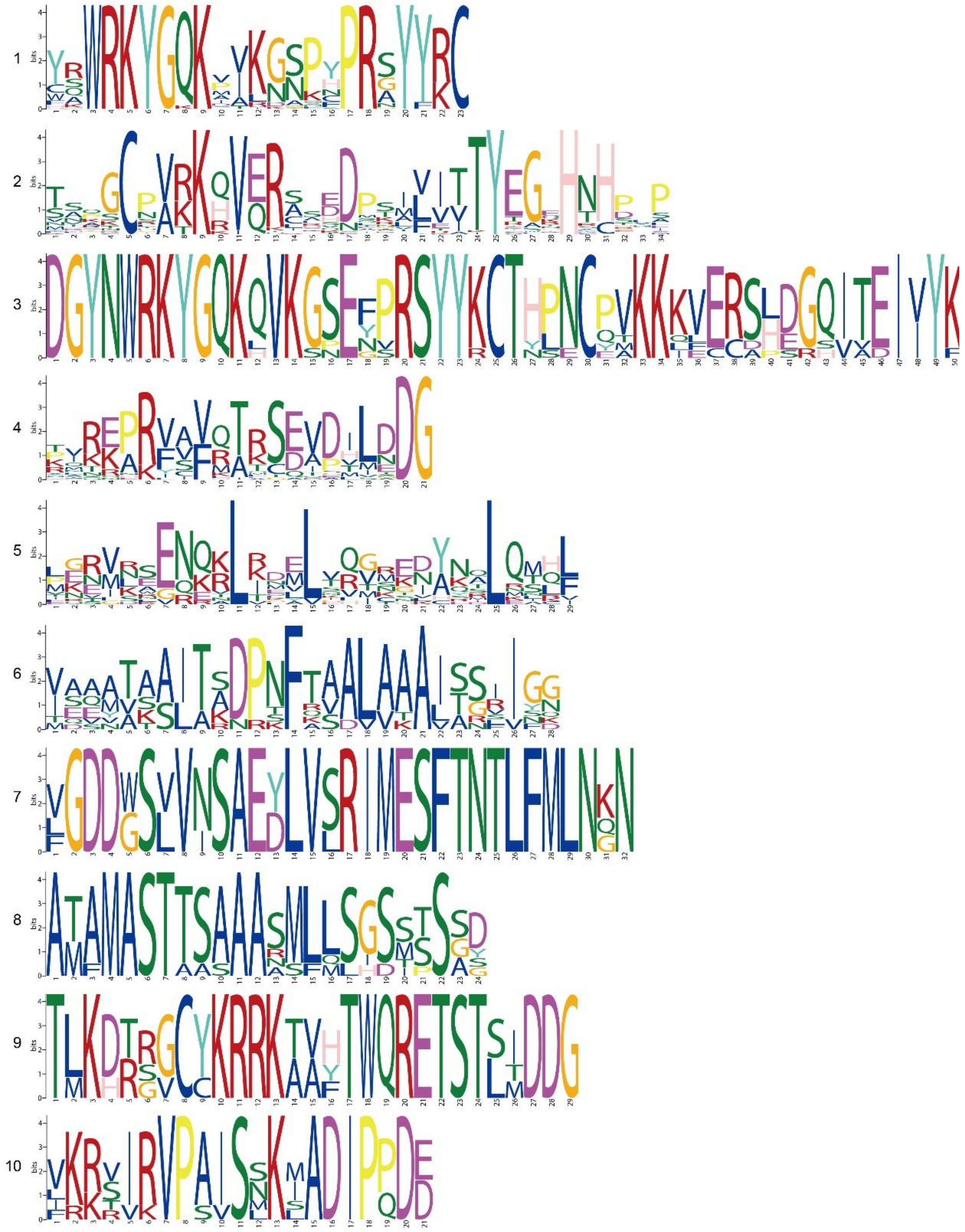

Figure S2. Sequence logos of the 10 conserved motifs identified in MnWRKY proteins. Conserved motif analysis was performed on 56 MnWRKY proteins using the MEME online tool with the number of motifs set to 10. The figure displays the amino acid conservation of each motif.

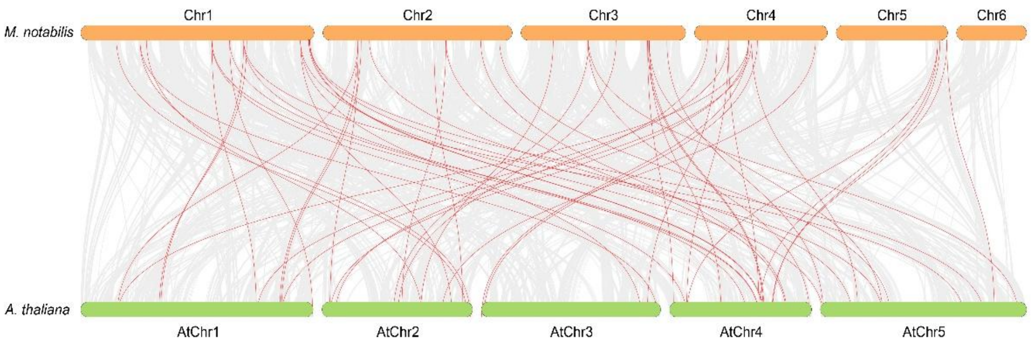

Supplementary Figure S3. Interspecies collinearity analysis of WRKY genes between *A. thaliana* and *M. notabilis*.

Note: Colored curved lines connect orthologous WRKY gene pairs identified by MCScanX. The six mulberry chromosomes (Chr1–Chr6) and five *Arabidopsis* chromosomes (AtChr1–AtChr5) are shown. This analysis was performed to examine the evolutionary conservation of WRKY genes between the two species.

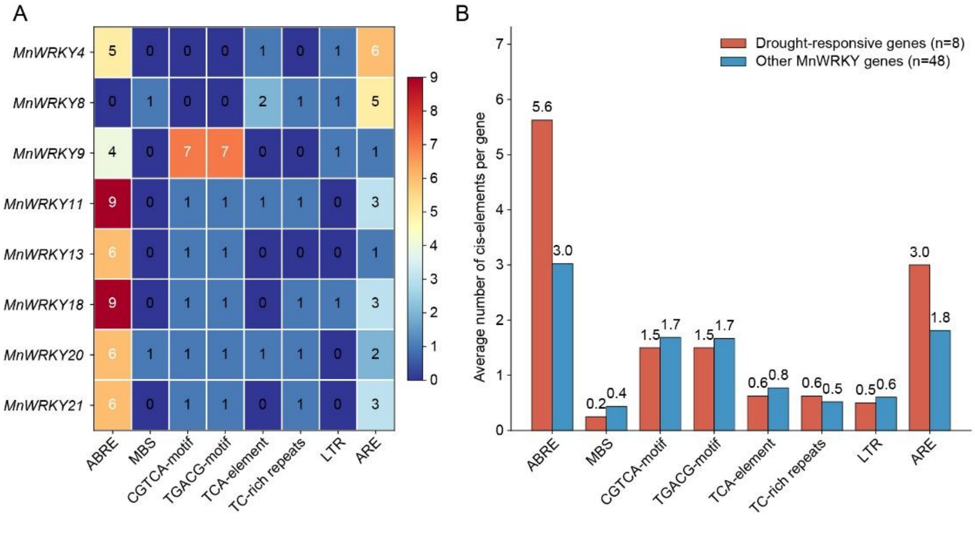

Supplementary Figure S4. Cis-element analysis of the eight consistently drought-responsive MnWRKY genes. (a) Heatmap showing the number of eight stress-related cis-elements in each gene. (b) Comparison of the average number of cis-elements between the eight drought-responsive genes and the remaining MnWRKY genes.

Note: Cis-elements were predicted using PlantCARE. The eight genes are *MnWRKY4*, *MnWRKY8*, *MnWRKY9*, *MnWRKY11*, *MnWRKY13*, *MnWRKY18*, *MnWRKY20*, and *MnWRKY21*. ABA, abscisic acid; MeJA, methyl jasmonate; SA, salicylic acid; LTR, low-temperature responsiveness; ARE, anaerobic induction.

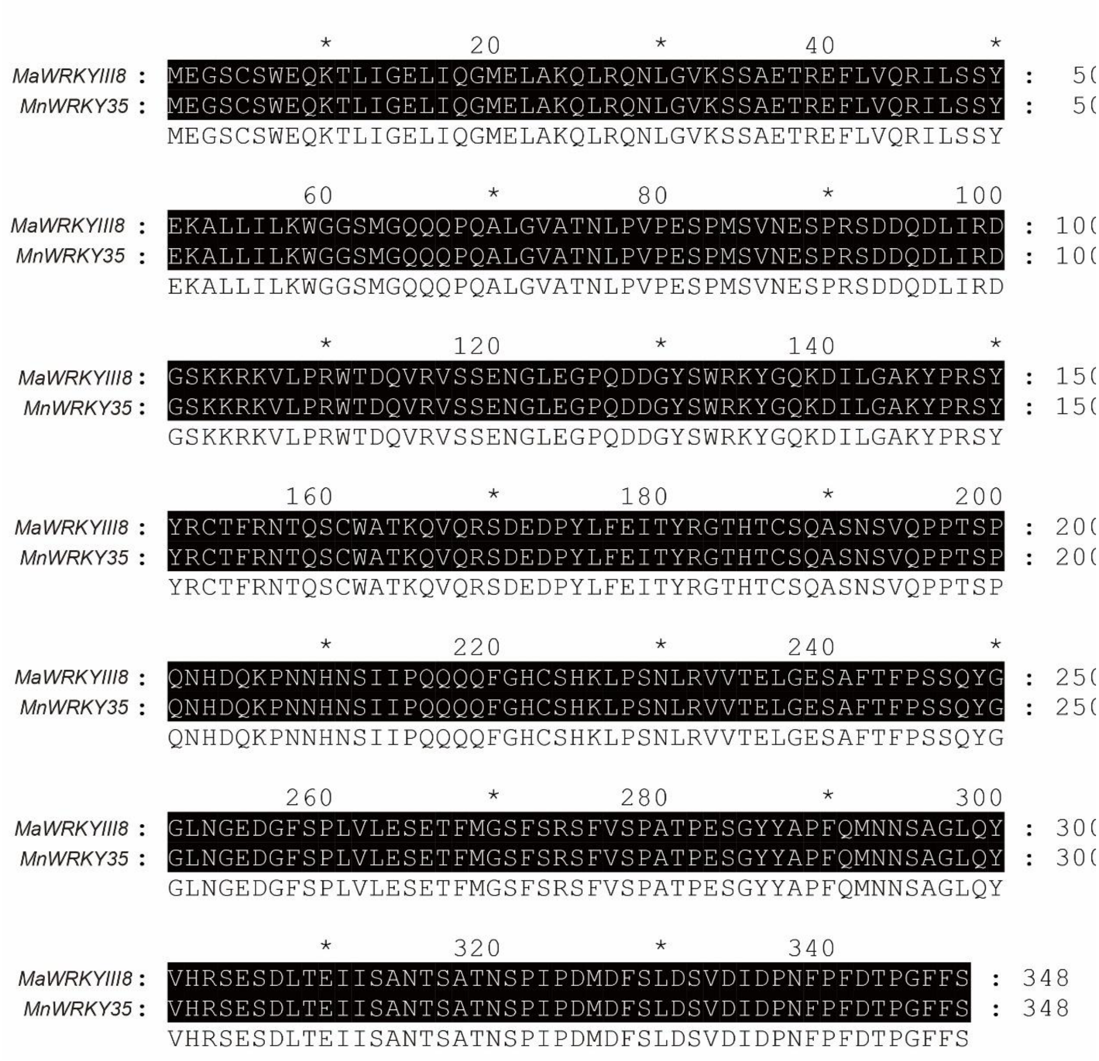

Supplementary Figure S5. Pairwise sequence alignment of MaWRKYIII8 (XP_010104968.1) and MnWRKY35 (MnotChr3G00179840.1) from *M. notabilis*.

Note: MaWRKYIII8 is the previously reported mulberry WRKY transcription factor 53. MnWRKY35 is the corresponding gene in the *M. notabilis* reference genome used in this study. This gene was previously named MnWRKY36 in the original submission and has been renamed MnWRKY35 after removing two non-WRKY candidates. The two sequences share 100% identity.

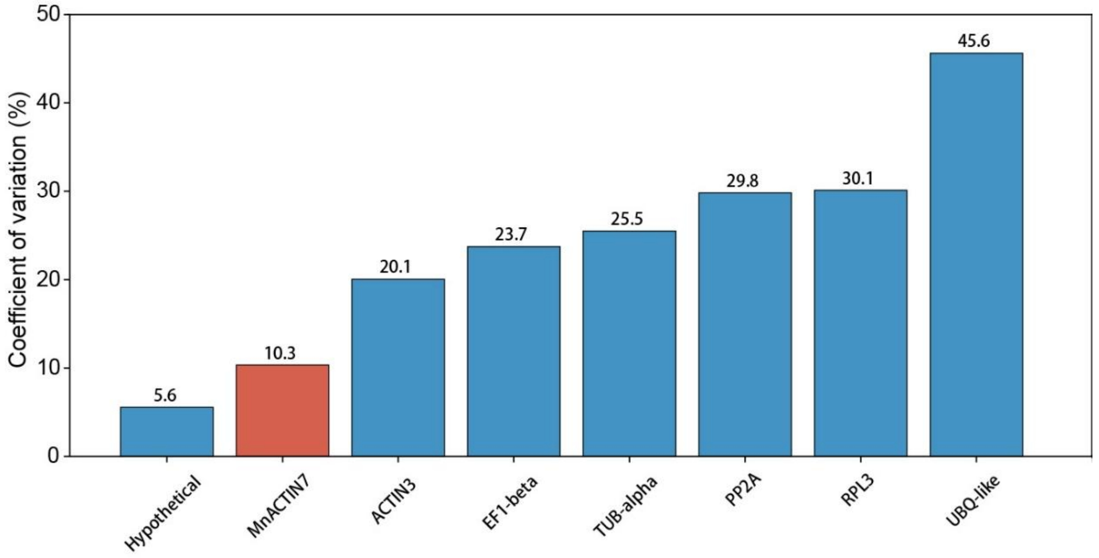

Supplementary Figure S6. Coefficient of variation (CV) of candidate reference genes under drought stress in mulberry.

Note: CV was calculated as the standard deviation divided by the mean of group means across drought treatments (0, 7, and 12 DPS). *MnACTIN7* is highlighted in red, and the other genes were previously validated as reference genes in mulberry. Genes are ordered by increasing CV.
